# Supplementary material for: Key Bacterial Taxa Differences Associated with Polypharmacy in Elderly Patients
Source: Microorganisms. 2025 Aug 12;13(8):1877. doi: 10.3390/microorganisms13081877 (PMC12388457; doi:10.3390/microorganisms13081877)
Supplement: Supplementary file 1 [file microorganisms-13-01877-s001.zip › microorganisms-3727560-supplementary.pdf]

Supplementary Table S1. Medication Frequencies of 2019 Group

Medication Name	n (%)
Albuterol Sulfate	3 (1.6)
Amlodipine	13 (7.1)
Amlodipine-benazepril	2 (1.1)
Amoxicillin	2 (1.1)
Anastrozole	1 (0.5)
Aspirin	9 (5.0)
Atorvastatin	17 (9.3)
Azelastine	2 (1.1)
Azithromycin	1 (0.5)
Basaglar KwikPen	1 (0.5)
Biotin	1 (0.5)
Bultabital-acetaminophen-caff	1 (0.5)
Carvedilol	6 (3.3)
Ceterizine	0
Chlorhexidine gluconate	5 (2.7)
Cyclobenzaprine	2 (1.1)
Enalapril maleate	2 (1.1)
Famotidine	2 (1.1)
Finasteride	2 (1.1)
Fluoxetine	3 (1.6)
Fluticasone propionate	1 (0.5)
Gabapentin	6 (3.3)
Glipizide	6 (3.3)
Hydrochlorothiazide	7 (3.8)
Hydrocodone-acetaminophen	3 (1.6)
Insulin	4 (2.2)
Jardiance	3 (1.6)
Latanoprost	2 (1.1)
Levothyroxine	3 (1.6)
Lisinopril	10 (5.5)
Loratadine	2 (1.1)
Losartan	7 (3.8)
Meloxicam	2 (1.1)
Metformin	14 (7.7)
Metoprolol succinate	4 (2.2)
Naproxen	4 (2.2)
Nifedipine	2 (1.1)
Omeprazole	4 (2.2)
Pantoprazole	3 (1.6)
Pregabalin	2 (1.1)
Propranolol	1 (0.5)
Simvastatin	5 (2.7)
Synthroid	2 (1.1)
Tadalafil	2 (1.1)

Tradjenta	2 (1.1)
Triamterene-hydrochlorothiazide	3 (1.6)
Valacyclovir	2 (1.1)
Ventolin HFA	1 (0.5)
Trazodone	0
Sertraline	0
Bupropion	0
Conazepam	0
Trazodone	0
Ventolin	0

Supplementary Table S2. Medication Frequencies of 2023 Group

Medication Name	n (%)
Albuterol Sulfate	9 (6.4)
Amlodipine	4 (2.9)
Amlodipine-benazepril	0
Amoxicillin	3 (2.1)
Anastrozole	1 (0.7)
Aspirin	13 (9.2)
Atorvastatin	7 (5.0)
Azelastine	0
Azithromycin	0
Basaglar KwikPen	0
Biotin	0
Bultabital-acetaminophen-caff	
Carvedilol	0
Ceterizine	2 (1.4)
Chlorhexidine gluconate	6 (4.3)
Cyclobenzaprine	0
Enalapril maleate	0
Famotidine	0
Finasteride	0
Fluoxetine	1 (0.7)
Fluticasone propionate	5 (3.6)
Gabapentin	4 (2.9)
Glipizide	0
Hydrochlorothiazide	10 (7.1)
Hydrocodone-acetaminophen	
Insulin	1 (0.7)
Jardiance	1 (0.7)
Latanoprost	1 (0.7)
Levothyroxine	2 (1.4)

Lisinopril	9 (6.4)
Loratadine	0
Losartan	8 (5.7)
Meloxicam	2 (1.4)
Metformin	8 (5.7)
Metoprolol succinate	3 (2.1)
Naproxen	5 (3.6)
Nifedipine	2 (1.4)
Omeprazole	2 (1.4)
Pantoprazole	3 (2.1)
Pregabalin	0
Propranolol	2 (1.4)
Simvastatin	2 (1.4)
Synthroid	1 (0.7)
Tadalafil	0
Tradjenta	0
Triamterene-hydrochlorothiazide	1 (0.7)
Valacyclovir	0
Ventolin HFA	3 (2.1)
Trazodone	3 (2.1)
Sertraline	3 (2.1)
Bupropion	4 (2.9)
Conazepam	4 (2.9)
Trazodone	3 (2.1)
Ventolin	2 (1.4)
